# Finding New Enzymes from Bacterial Physiology: A Successful Approach Illustrated by the Detection of Novel Oxidases in *Marinomonas mediterranea*

**DOI:** 10.3390/md8030519

**Published:** 2010-03-05

**Authors:** Antonio Sanchez-Amat, Francisco Solano, Patricia Lucas-Elío

**Affiliations:** 1 Department of Genetics and Microbiology, Faculty of Biology, University of Murcia, Campus de Espinardo, Murcia 30100, Spain; E-Mail: patlucel@um.es; 2 Department of Biochemistry and Molecular Biology B and Immunology, School of Medicine, University of Murcia, Murcia 30100, Spain; E-Mail: psolano@um.es

**Keywords:** Marinomonas, laccase, lysine oxidase, tyrosinase, melanin

## Abstract

The identification and study of marine microorganisms with unique physiological traits can be a very powerful tool discovering novel enzymes of possible biotechnological interest. This approach can complement the enormous amount of data concerning gene diversity in marine environments offered by metagenomic analysis, and can help to place the activities associated with those sequences in the context of microbial cellular metabolism and physiology. Accordingly, the detection and isolation of microorganisms that may be a good source of enzymes is of great importance. *Marinomonas mediterranea*, for example, has proven to be one such useful microorganism. This Gram-negative marine bacterium was first selected because of the unusually high amounts of melanins synthesized in media containing the amino acid l-tyrosine. The study of its molecular biology has allowed the cloning of several genes encoding oxidases of biotechnological interest, particularly in white and red biotechnology. Characterization of the operon encoding the tyrosinase responsible for melanin synthesis revealed that a second gene in that operon encodes a protein, PpoB2, which is involved in copper transfer to tyrosinase. This finding made PpoB2 the first protein in the COG5486 group to which a physiological role has been assigned. Another enzyme of interest described in *M. mediterranea* is a multicopper oxidase encoding a membrane-associated enzyme that shows oxidative activity on a wide range of substrates typical of both laccases and tyrosinases. Finally, an enzyme very specific for l-lysine, which oxidises this amino acid in epsilon position and that has received a new EC number (1.4.3.20), has also been described for *M. mediterranea*. Overall, the studies carried out on this bacterium illustrate the power of exploring the physiology of selected microorganisms to discover novel enzymes of biotechnological relevance.

## 1. Introduction

It is widely accepted that there is an enormous range of microbial diversity not yet accessed or explored that might serve for possible novel biotechnological applications. This is particularly true in the case of marine environments, as revealed by recent metagenomic analysis showing the enormous range of novel proteins that can be predicted [1]. Since the discovery of the possibility of cloning DNA directly from the environment, this approach has been used to explore metagenomic libraries for novel metabolic routes and enzymes in a wide range of environments, including marine samples [2].

In addition to the interest in exploiting marine biodiversity using culture-free methods, there is a huge diversity of culturable marine microorganisms belonging to different taxonomic groups that have not been characterized in depth. This is true, for example, in the case of microorganisms living in biofilms on the surface of higher organisms. In such environments, microorganisms develop complex interactions among themselves and with the host, which can determine the expression of unique characteristics of possible biotechnological interest [3]. Marine plants and invertebrates are examples of higher organisms with a rich community of associated microorganisms of great potential biotechnological interest, particularly for the production of secondary metabolites [4]. Marine surface-associated microorganisms may also be of interest for the production of enzymes. For example, the genome sequence of the epiphytic marine bacterium *Saccharophagus degradans* strain 2–40 has revealed the presence of numerous genes involved in the degradation of polysaccharides [5]. This observation led to the cloning and expression of one of these enzymes with biotechnological interest [6].

**Figure 1:**
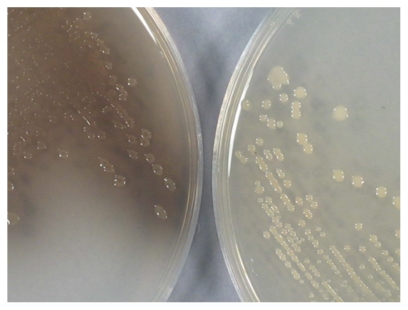
Pigmentation of *M. mediterranea* MMB-1 (left) and mutant strain T103 (right) in Marine Agar 2216 after three days of incubation at 25 °C.

In our opinion, the possibility of finding novel activities could be increased by the study of selected strains of different taxonomic groups showing unique characteristics. These data would also be of interest for understanding the mechanism of adaptation of microbial strains to different environments. The marine bacterium *Marinomonas mediterranea*, isolated by our group, is an example of a microorganism with unique characteristics. It belongs to a genus in which no other members have been studied in depth, with the exception of *Marinomonas* sp. strain MWYL1, which has been studied in relation to the generation of the gas dimethylsulphide [7]. *M. mediterranea* MMB-1 was initially selected from a seawater sample for its unique characteristic of showing a dark pigmentation in complex media, as shown in Figure 1 [8].

**Figure 2:**
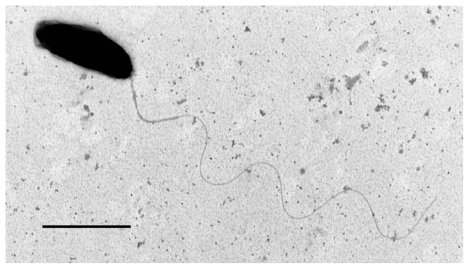
Electron micrograph of *M. mediterranea* IVIA-PO-186 negatively stained with uranyl acetate. Bar = 1 μm.

The taxonomic characterization of strain MMB-1 revealed that it belongs to a new species in the genus *Marinomonas*, and the species *M. mediterranea* was created [9]. Later, it was shown that this species can be isolated from the surface of the seagrass *Posidonia oceanica* [10]. *M. mediterranea* strains are Gram-negative rods with a single polar flagellum (Figure 2). All the strains isolated belonging to this species share the characteristic of producing dark pigments, unlike other *Marinomonas* species described so far [10].

As will be discussed in the next sections, *M. mediterranea* has proven to be an excellent source of oxidative enzymes, including the tyrosinase responsible for pigmentation, a multicopper oxidase with laccase activity, and a novel lysine oxidase. So far, it is the only microorganism described showing such a combination of enzymes of potential biotechnological interest in many different fields particularly in white and red biotechnology. Moreover, data from our laboratory suggest that additional oxidative enzymes are synthesized by this strain (Lucas-Elío, P and Sanchez-Amat, A., unpublished results).

## 2. Melanin, Melanogenesis and Tyrosinase Activity

### 2.1. Types and synthesis of melanin pigments

Melanin pigmentation is one of the most ancient and universal processes of living organisms. Melanins are a group of pigments derived from the hydroxylation, oxidation and polymerization of phenolic compounds. Numerous studies and reviews are available on the genetics, synthesis, properties, roles, and biomedical aspects of melanins in human and higher animals [11], but less is known about microbial melanins [12], and less still about melanins in marine microorganisms.

According to the precursors, biosynthetic pathway and final composition of the polymer, there are basically three types of melanins: eumelanins, pheomelanins and allomelanins [13]. Eumelanins and pheomelanins are more abundant in the skin and hair of animals, although they can also be found in the microbial kingdom. Both are produced by the Raper-Mason pathway by o-hydroxylation of the amino acid l-tyrosine *via* l-dopa and l-dopaquinone. It has sometimes been proposed that l-phenylalanine should also be considered a melanin precursor, since phenylalanine hydroxylase can convert this amino acid into l-tyrosine [14]. However, a number of data indicate that the contribution of phenylalanine hydroxylase activity to melanogenesis in most species is negligible, although occasionally this reaction has been questioned and reconsidered [15]. In any case, the putative hydroxylation of l-phenylalanine simply contributes to increasing the l-tyrosine pool inside melanogenic cells to be later converted into l-dopaquinone. Depending of both environmental and genetic conditions, l-dopaquinone can undergo (i) an internal cyclation through its amino group on the side chain to form an indole ring (the structural unit of eumelanins) or (ii) the addition a thiol-compound (usually cysteine or glutathione) to yield cysteinyldopa (the structural unit of pheomelanins). Undefined polymerization of the respective structural units leads to the final pigment. As a result of this process, eumelanins are larger and darker (brown to black) than pheomelanins (yellow to red sulphur-containing pigments).

Allomelanins are the least studied, but most heterogeneous group of melanin pigments. They are usually dark and are generally characterized by the absence of nitrogen, as they are formed from catechols (mostly in plants), from 4-hydroxyphenylacetate (some bacteria) or from dihydroxynaphtalene (DHN) *via* the pentaketide pathway (mostly in microorganisms). In rare cases, they contain units of metadiphenols as opposed to the usual ortho and para positions of eumelanins [16]. There are also reddish-colored allomelanins, such as those derived from gamma-l-glutaminyl-4-hydroxybenzene (mushrooms) or from homogentisic acid (different types of cells including fungi and bacteria). The latter are sometimes called pyomelanin [17,18].

Melanins are usually described as pigments that protect against a number of environmental stress conditions, and melanogenesis as an adaptation response against those conditions. In animals, cutaneous melanin is mostly considered a photoprotective pigment against UV radiation, although sometimes it is related to camouflage, ornamentation for sexual attraction, *etc*. In addition to its UV absorbing properties, melanin also offers protection as a cellular scavenger against free radicals, ROS, drugs, oxidants and xenobiotics. The extrapolation to microorganisms of these protective roles, especially the photoprotective role, is controversial, but it has been reported that some microorganisms synthesize melanin in response to other stress conditions, such as high temperatures, starvation or hyperosmotic media [19]. In pathogenic microorganisms melanins have been considered as important virulence factors [20,21].

Alternative roles have been proposed for melanins in marine bacteria. One of the most interesting proposals, based on the fact that melanins contain quinone/quinol groups, is that they could act as electron acceptors in anaerobic respiration, replacing oxygen in the last step of the respiratory chain [22,23]. The redox properties of the polymer could also serve as reductor of insoluble oxidized metal oxides, such as iron or manganese oxides, to the divalent soluble state. In mineralized marine deposits, the availability of such oligoelements in soluble form is limited, and melanin production might be a mechanism of evolutionary adaptation to mobilize those cations from the bottom of the sea and to store them in melanized cells [12].

### 2.2. Enzymes involved in melanin synthesis. Polyphenol oxidases, tyrosinases and catechol oxidases

In spite of the complexity and multistep pathway leading to the formation of the melanin polymer, in the presence of oxygen most of the steps are spontaneous oxidations, and the enzymatic regulation only occurs in the initial stage(s) of the pathway. Since the initial precursors of melanin are phenols, the key enzyme was generically named polyphenol oxidase (PPO). Almost all PPOs related to melanogenesis have an active site characterized by a pair of antiferromagnetically coupled copper ions, CuA and CuB, which are coordinated by six histidine residues [24,25]. It should be noted that living cells contain other non-copper enzymatic systems able to catalyze phenol hydroxylation, such as cytochrome P450, 4-hydroxyphenylacetate or 4-hydroxyphenylpyruvate hydroxylase. These, too, may be considered PPOs, and, at appropriate conditions, they may well produce melanins, but as laccases (see section 3.1), they usually do not play a central role in physiological melanin formation.

Although PPO is the most general name for the family, the name tyrosinase was early introduced and is widely used, since l-tyrosine is one of the most common phenolic precursors [26,27]. Tyrosinases (EC 1.14.18.1) are ubiquitously distributed in all types of cells, from higher animals to bacteria [28,29]. They bind one molecule of atmospheric oxygen at the dicopper active site to catalyze two different kinds of enzymatic reactions (i) the *ortho*-hydroxylation of l-tyrosine/monophenols (tyrosine hydroxylase or cresolase activity) and (ii) the oxidation of l-dopa/*o*-diphenols to *o*-quinones (dopa oxidase, catecholase or catechol oxidase activity). Using a monophenol as the initial substrate, both reactions occur consecutively to yield the corresponding *o*-quinone. However, the catalytic mechanism of the first reaction is rather complicated and shows an unusual “lag period” (a catalytically silent period before product appearance), whose time length varies depending on the enzyme source and conditions [30]. This lag period in tyrosine hydroxylation can be shortened, to an extent that also depends on the source of the tyrosinase, by the presence of an *o*-diphenol or some divalent ions, such as Fe(II). The molecular mechanisms accounting for this feature are beyond the scope of the present review. In practice, some tyrosinases, *i.e.*, mushroom tyrosinase, show a very short lag period (several seconds), while others show long lag periods (hours to days), meaning that they lose practically all their tyrosine hydroxylase activity. When the lag period is so long, tyrosinases can be considered enzymes with only *o*-diphenol (catechol) oxidase activity and so they are named catechol oxidases (EC 1.10.3.1). Catechol oxidases are quite usual in plants, so in fruits most PPOs, which are responsible for the fruit browning after injury or maturation, are classified as catechol oxidases [31]. In terms of active site structure and other features, such as CuA and CuB binding sites with essential histidine residues, *etc*., the difference between “pure” catechol oxidase and tyrosinase with a very long lag period is an unresolved issue. Nevertheless, both have long had a distinct enzyme number assigned to them due to the catalytic differences they show. Both catechol oxidase and tyrosinase enzymes are able to form melanin, but the initial precursor is, respectively, an o-diphenol or a monophenol.

### 2.3. Biotechnological applications of melanin and tyrosinases

Different bioapplications have been proposed in the literature for both the melanin pigment as well as for the oxidases involved in melanogenesis. Eumelanins, which have the best photoprotective properties, have been added to sunscreen lotions [32]. However, the dark colour of their emulsions makes them commercially unattractive, which has hampered their use. Despite this, some lotions, such as MelanSol®, are available on the market. The most usual sources for these melanins are synthetic dopa-melanin, mammalian hair and cuttlefish ink, although the use of bacterial-derived melanin as photoprotectors in active sunscreen has recently been reported [33].

Melanins have a polymeric organic structure with some energy conversion ability and semiconductor properties, which provides them with a possible role in photoelectrochemical applications and solar energy conversion. Some attempts have been made to use them as components of solar panel energy-converters [34,35]. As before, the exploration of bacterial melanins, as alternative to those from mammalian hair, might be interesting, as the fine structure of melanin from different sources differs, and this might be relevant for identifying the most appropriate ones for specific applications.

Melanin pigments are easy to detect as shown in Figure 1, a property that has been used in certain molecular applications. For example, vectors for the study of transcriptional regulation and promoter regions have been developed using the *Streptomyces* tyrosinase as reporter [36]. Melanin synthesis has also been used to develop a method for high-throughput screening of tyrosine secretion in *E. coli* expressing a recombinant tyrosinase [37].

In regards to the possible biotechnological applications of tyrosinases, the most widely accepted one is as a biosensor for the accurate and sensitive determination of phenolics in different industrial fluids, ranging from wine [38] to waste residues containing polluting phenols [39]. Tyrosinases are particularly useful proteins for such applications as they are resistant enzymes and have relatively long half-lives after immobilization by different chemical methods and in different materials, including silicone-grease [40] and alumina-gel [41].

Some tyrosinases are able to oxidize not only free l-tyrosine, but also the side chain of tyrosine residues in peptides and proteins to form dopa and dopaquinone residues on the polypeptidic chain. Marine adhesive proteins secreted by mussels and other invertebrates insolubilize and adhere to the surfaces of a variety of materials by means of a quinone cross-linking insolubilization reaction catalyzed by a tyrosinase [42–44]. Bearing this in mind, tyrosinase might also be useful for the preparation of adhesive glues in aqueous environments or on wet surfaces, alone [45] or even mixed with the adhesive gecko keratins in a new material named “geckel” (from gecko and mussel).

### 2.4. Tyrosinases and melanin-producing marine microorganisms

Melanin synthesis and tyrosinase activity have been reported only in a few marine microorganisms but particularly in bacteria. *Vibrio cholerae* was one of the first described melanin-pigmented bacterial strains, although this strain does not have a true tyrosinase [46,47] and the synthesized pigment is a type of allomelanin derived from homogentisic acid, as a consequence of alterations in the tyrosine catabolism rather than with the existence of a melanin synthetic pathway [17].

*Saccharophagus degradans* 2–40 is an epiphytic bacterium in which melanin synthesis and a tyrosinase have been reported [48]. Another melanin-pigmented bacterium is *Alteromonas nigrifaciens*, although the characterization of its enzymatic system has not been carried out [49]. Finally and very recently, strong tyrosinase activity has been described in *Cellulophaga tyrosinoxydans* sp. nov. [50]. The pigment synthesized by this strain is yellow, suggesting a pheomelanin nature.

Interestingly, in the *Roseobacter* clade, which is a significant component of marine microbiota, particularly of the surface microbiota associated to algae, the synthesis of antimicrobial compounds has been associated with brown pigmentation. However, the exact nature of the pigment remains to be determined [51].

An important group of melanin-synthesizing microorganisms are the actinomycetes, particularly the genus *Streptomyces*, from which most of the compounds with known biological activity have been isolated. *Streptomyces* strains isolated from marine environments share the characteristics of being able to synthesize melanins [52] and of being an excellent source of novel compounds [53]. The genus *Streptomyces* is the most usual source of prokaryotic tyrosinases for genetic, structural and spectroscopical studies. The *Streptomyces* tyrosinase is a monomeric protein with a low molecular mass, around 30 kDa, that is secreted to the surrounding medium, where it is involved in extracellular melanin production. In principle, *Streptomyces* are a good source of soluble tyrosinases due to their high specific activity, stability and the ease with which the enzyme can be purified. Most of *Streptomyces* species harbor a *melC* operon, in which *melC2* encodes the extracellular tyrosinase and *melC1* encodes a helper protein essential for the correct expression of the enzyme since it is involved in the copper transfer to apotyrosinase to yield the active enzyme [54]. Moreover, it has been found that the *Streptomyces* chromosome contains a second *melC*-homologous operon (*melD*). The data obtained suggest that *melC* and *melD* have divergently evolved toward different functions. Unlike tyrosinase (MelC2), MelD2 is not secreted, and has a narrower substrate spectrum. Deletion of *melD* causes increased sensitivity to several phenolics that are substrates of MelD2 [55].

Focusing on melanin-producing marine bacteria, the most illustrative example is undoubtedly *M. mediterranea*, a bacterium that is able to form black eumelanin from l-tyrosine [8] and which expresses two copper enzymes with PPO activity. The enzyme responsible for melanin formation is a true tyrosinase (PpoB1) [25] and the second PPO is PpoA, a multipotent membrane-bound laccase [56]. PpoB1 is part of an operon with a second gene that encodes the protein named PpoB2, which is involved in the specific copper transfer to PpoB1, but not to other copper oxidases such as PpoA [57]. Interestingly, the structure and mechanism of PpoB2 differs from the chaperones encoded in the *Streptomyces* operon. PpoB2 shows similarities with the COG5486 group encoding putative transmembrane metal binding proteins [58], and, to the best of our knowledge, is the first case of this type of protein involved in copper transfer in bacteria. The existence of this melanin-formation system, PpoB, with its novel type of copper-transfer chaperone, and the multipotency in regards to substrate specificity of PpoA, make *M. mediterranea* a very interesting model for studies concerning bacterial copper-oxidases.

## 3. Multicopper Oxidases (MCOs) and Laccases

### 3.1. General characteristics of MCOs

Blue multicopper protein oxidases (MCO) are proteins characterized by the presence of at least four copper ions. According to their spectroscopic properties, they are classified as copper type 1 (T1), copper T2 and a pair of copper T3 that are coupled and silent when studied by electron paramagnetic resonance spectroscopy. T2 and T3 form a trinuclear copper center [59]. The MCO are related to other multidomain proteins, whose redox properties are based on the presence of copper ions. For all these proteins, a common evolutionary origin has been proposed [60]. A wide range of proteins from different organisms belong to the group of MCO, including ascorbate oxidase, ceruloplasmin and laccases [61]. Laccases (E.C. 1.10.3.2) are oxidases able to oxidize phenols, mainly p-diphenol and methoxy-substituted monophenols, although they can also oxidize other non-phenolic compounds such as aromatic amines, 2-2′-azino-bis(3-ethylbenzothizoline-6-sulphonic acid (ABTS), dyes and inorganic ions. During oxidation by laccases, oxygen is reduced, through the transfer of four electrons, to water. Because of their capacity to oxidize phenols, laccases can be included in the group of PPOs (section 2.2). A distinctive feature of tyrosinases and laccases is that the latter is considered not to oxidize l-tyrosine [62].

The use of the term laccase is sometimes confusing since some authors use this name only for those enzymes synthesized by plants and fungi showing high activity on phenolic compounds, while others use this term in a broader sense to refer to any enzyme able to oxidize a laccase substrate. However, it is important to bear in mind that the broad substrate range of MCO means that they can oxidize different compounds. For instance, in several *Pseudomonas* strains CumA, a MCO involved in manganese oxidation, has been detected. This MCO is not only able to oxidize Mn but also oxidizes the laccase substrate ABTS [63]. In turn, a fungal enzyme, first described as a laccase, was lately found to also oxidize Mn [64]. Accordingly, the term laccase sometimes makes reference to the enzymatic activity of a protein, although proteins with such activity can play different physiological roles or oxidize other kinds of compounds.

Most laccases studied so far have been isolated from plants and fungi [62,65]. In fact, the name laccase derives from the first description of an enzyme of this group in the lacquer tree [66]. In plants, laccase activity has been associated to lignosynthesis [67]. In contrast, fungal laccases produced by white rot fungi show a lignolytic capacity, which is increased by the use of small molecules that act as mediators [68]. In addition, fungal laccases may also be involved in pigment synthesis and are considered important factors in pathogenesis [69].

MCO with laccase activity has also been detected in bacteria. The first report was in the bacterium *Azospirillum lipoferum*, in which it was associated to pigment synthesis [70]. Next, the activity was reported in the marine bacterium *M. mediterranea* [8], *E. coli* [71] and *Bacillus subtilis* [72], and it is now accepted that MCOs are widely distributed in bacteria, where they may be related to different physiological processes such as copper resistance [73].

Interestingly, the first archaeal laccase was recently characterized from *Haloferax volcanii*. The glycosylated enzyme shows strong resistance to high temperatures and high salt concentrations, which will surely be of interest for a variety of applications such as the detoxification of lignin from lignocellulosic material for ethanol production [74].

### 3.2. Biotechnological interest of laccases

Their high capacity to oxidize different substrates, and the fact that they use oxygen as electron acceptor and do not require cofactors, make laccases interesting enzymes in industrial processes [75,76]. In fact, at least two applications of these enzymes are in commercial use. The enzyme Suberase® is used for the removal of free phenols from corks, avoiding the potential problem of wine contamination by these chemicals. Laccases are also commercially available in the textile industry for denim bleaching.

Since laccases are able to degrade lignin, there is a growing interest in using this characteristic in processes such as the pulp and paper industry and the degradation of lignocellulosic material for the generation of biofuels [68,77].

Several studies have addressed the use of laccases for removing contaminant dyes [78]. In addition, laccases and tyrosinases have also been evaluated in the food industry; for example in baking, as cross-linking agents for the modification of the properties of bread [79]. Laccases can be used as biosensors of phenols in different samples, such as olive oil mill wastewater [80] and, in combination with a tyrosinase, in beer [81]. Another interesting application of laccases is their use as biocatalysts in the modification and generation of novel compounds with biological activities: for example, the modification of beta-lactamic antibiotics [82], of corollosporine, which is isolated from the marine fungus *Corollospora maritima* [83] and of curcuphenol, a metabolite derived from the sponge *Didiscus aceratus* [84].

Depending on the source of the enzyme, the range of substrates and other enzymatic properties might show some differences. For example, bacterial laccases tend to be active at basic pH, while, in general, fungal enzymes are active at acidic pH [78]. Taking into consideration the wide range of applications of laccases, exploration of novel microbial sources of these enzymes will be of interest.

### 3.3. MCOs in marine microorganisms

Several microorganisms expressing laccase activity have been detected in seawater samples. Laccase-producing marine fungi have been isolated from environments rich in lignocellulosic material such as mangroves, from which a fungus expressing a thermostable and metal-resistant laccase with potential interest for the bioremediation of dye-contaminated waters and for the degradation of lignin in sugarcane basse pulp has been isolated [85].

Regarding marine bacteria, several studies have reported the presence of MCOs with Mn oxidase activity, which, as explained above, also show laccase activity. The phylogenetic diversity of these bacteria is high, and includes Gram-positive *Bacillus* strains [86], and Gram-negative bacteria [63]. Moreover, analysis of the genome of one of these alphaproteobacteria also revealed the presence of additional MCOs and other proteins involved in Cu homeostasis [87].

Another marine bacterium which expresses an enzyme with laccase activity is *Pseudoalteromonas haloplanktis* [88]. The gene encoding this enzyme shows similarity with MCOs involved in copper resistance and, accordingly, it has been named *PhCopA*. The protein is induced by the presence of copper in the medium and its capacity to degrade catechol allows a recombinant strain expressing toluene-o-xylene monooxygenase to grow using phenols as sole carbon source [88]. In the epiphyte *S. degradans* 2–40, mentioned above as expressing a tyrosinase, an MCO with laccase activity very similar to the *M. mediterranea* laccase PpoA described below was also described [9].

*M. mediterranea* was the first marine bacterium, and one of the first bacteria in general, in which laccase activity was detected [8]. This protein is membrane-associated and analysis of its enzymatic characteristics indicated that it is able to oxidize substrates characteristic of both laccases and tyrosinases, which is an unusual property for a laccase [89]. In the case of *M. mediterranea* several molecular techniques have been developed allowing the gene *ppoA*, which encodes the laccase, to be cloned by transposon mutagenesis [90]. Analysis of the sequence revealed that, in addition to the characteristic copper centers of laccases, it contained another two potential sites involved in copper binding [56]. The recombinant expression of the full protein or of fragments of it, suggested that all copper centers are important for the laccase activity of PpoA [56].

The physiological role of PpoA remains to be determined, but results obtained so far indicate that it could be different to the role of other bacterial MCOs described. For instance, it is not involved in Mn metabolism (C. Francis, personal communication). Other data indicate that it is not involved in Cu resistance, as suggested by the fact that it is not induced by copper [89]. Also, unlike fungal PPOs, no evidences has been found to show that it is induced by phenols such as xylidine [89]. As regards potential biotechnological interest of PpoA, this enzyme shows high activity on phenolic compounds, and it has been seen to be more tolerant to basic pH and halide than the fungal enzymes [91].

## 4. l-Amino Acid Oxidases

### 4.1. General aspects about l-amino acid oxidases

l-Amino acid oxidases (LAOs) are generally considered to be flavoenzymes which catalyze the oxidative deamination of an l-amino acid to its α-keto acid (EC 1.4.3.2). Just as the above mentioned PPOs, these enzymes belong to the family of oxidoreductases. The reaction catalyzed by LAOs (1) requires the presence of oxygen as the electron acceptor and leads to hydrogen peroxide and ammonium as products. Hydrogen peroxide released from the reaction plays a decisive role in the bactericidal effect that has been described for some of these enzymes [92].

(1) $$\text{L}-\text{amino acid }({\text{RCHNH}}_{2}\text{COOH})+{\text{H}}_{2}\text{O}+{\text{O}}_{2}⇌\alpha -\text{keto acid }(\text{RCOCOOH})+{\text{NH}}_{3}+{\text{H}}_{2}{\text{O}}_{2}$$

Oxidase activity is commonly exerted on a wide range of amino acids, although some LAOs show a certain degree of specificity for a given amino acid. Such is the case with the glycine oxidase of *Bacillus subtilis* [93], l-glutamate oxidase of *Streptomyces* sp. [94] or l-lysine oxidase from fungi, particularly of the genus *Trichoderma*, the last of which has been studied in relation to its antitumor properties [95,96].

LAOs have received considerable attention as components of snake venoms, in which they contribute to their anti-protozoal, bactericidal, anti-viral and pro-apoptotic effects [97], but similar effects are also found in LAOs of mammalian leukocytes and milk [98].

In contrast to that of earth snakes, sea snake venoms show little LAO activity [99]. The best studied LAO in the sea come from some marine mollusks like the sea hares of the family *Aplysiidae*, which use LAOs as defensive compounds against predators. In order to prevent autotoxicity, the enzyme and its substrate, mainly l-lysine, are compartmentalized in two different glands. The mixing of both compounds only takes places upon predatory attack when the venom is pumped out of a syphon towards the attacker [100]. Other gastropods have LAOs on their surfaces, for example the albumen gland packaging the egg masses, to prevent colonization by microorganisms, [101], or to prevent infections of the skin after an attack [102]. The tumoricidal activity of these LAOs from marine gastropods and their resistance to degradation has been the subject of several studies [103].

A similar defensive role is found in the gametophytes of the red alga *Chondrus crispus*, which have an increased resistance to the endophytic green algal pathogen *Acrechaetae operculata*. This advantage is due to an l-asparagine oxidase induced in *C. crispus* in response to the l-Asn secreted by the pathogen after the first interaction. The hydrogen peroxide released in the reaction turns out to be more toxic for the endophyte than for the red seaweed [104].

The best characterized bacterial LAO is that expressed by *Rhodococcus opacus* which shows abroad substrate specificity. This enzyme has been expressed heterologously and its dimeric structure has been described [105,106]. LAOs are continuously being detected from diverse bacterial genera and different physiological functions have been proposed for them. Some of them are induced when amino acids are needed as nitrogen source [107]. Others seem to have a role in the competition between species, as observed by the production of hydrogen peroxide by *Streptococcus oligofermentans* in the cariogenic biofilm [108]. For the cyanobacteria *Anacystis nidulans* a function in electron transfer has been proposed at the thylakoid membrane [109]. In addition, α-keto acids, the products of the reaction, seem to be able to function as siderophores, forming complexes with iron, as in *Proteus mirabilis* [110].

In eukaryotic microorganisms, the main physiological function proposed for LAOs is related to the utilization of amino acids as nitrogen source. The green alga *Chlamydomonas reinhardtii* is able to use a wide range of amino acids as nitrogen source due to the LAO activity secreted into the medium [111]. In the filamentous fungus *Aspergillus nidulans* an LAO is responsible for the catabolism of most amino acids, and is specifically induced under nitrogen limiting conditions [112]. Similarly, *Neurospora cra*ss*a* requires nitrogen catabolite derepression to be able to use amino acids as nitrogen source through an LAO [113].

### 4.2. Biotechnological interest of LAOs

The most interesting aspect about LAOs is the possible biomedical use of their antitumoral and apoptotic properties. Among these enzymes, fungal l-lysine oxidases have been tested “*in vivo*” on several tumors with good results [114]. In addition, l-amino acid oxidases from snake venoms are widely studied because of their apoptotic inducing properties [115] or even as a coadjuvant in chemotherapy, as it has been demonstrated that low levels of amino acids in plasma increase the blood-tumor tissue transfer of antitumor drugs such as melphalan [116].

Another field of interest of LAOs is their use as biosensors. In most such applications, the LAOs are coupled with a detector of the hydrogen peroxide produced [117] or the oxygen consumed [118]. Food industry has a special interest for these biosensors, as the quality of many products is determined by their l-amino acid content [119,120]. In this sense, the determination of l-lysine is of great importance, as it constitutes the limiting amino acid in wheat, rice and maize, to ensure the nutritional quality of these products. The LAO of the black rockfish *Sebastes schlegeli* has been proposed for sensing l-lysine [121].

LAOs are also used in biotransformations such as in the separation of enantiomers from racemic mixtures of amino acids or the industrial production of keto-acids [122,123]. In a different application, the l-amino acid oxidase from *Rhodococcus* sp. has proved to be useful in the bioconversion and synthesis of aminoadipic derivatives, which are important as precursors for β-lactam antibiotics [124]. Other LAOs are being studied and characterized for their role in food processing, as in the enzymatic treatment for dough to reduce its viscosity and provide a better workability [125].

### 4.3. l-Amino acid oxidases in marine microorganisms

Marine phytoplankton shows cell-surface LAO activity under nitrogen-limited conditions. Only the ammonium released from the reaction is transported inside the cell, while the α-keto acid remains in the external medium [126]. Phytoplankton has been proposed to play a role in remineralizing nitrogen in the sea by means of this activity [127].

As for marine bacteria, there are few examples describing LAO activity. For example, this activity has been reported for the commensal microbiota of mackerel [128]. On the other hand, a tryptophan oxidase activity has been described as playing a role in the synthesis of the pigment violacein in the free living marine bacterium *Chromobacterium violaceum* [129]. Other violacein pigmented marine bacteria, such as *Pseudoalteromonas luteoviolacea*, have shown broad range l-amino acid oxidase activity [130].

l-lysine oxidase activity was first described in *M. mediterranea* as an antimicrobial protein named marinocine [131]. Later, the enzymatic activity of marinocine was elucidated [132,133]. The enzyme has a high specificity for l-lysine, which is oxidized in the presence of molecular oxygen in epsilon position to generate 2-aminoadipic semialdehyde, ammonium and hydrogen peroxide (2). This activity had never been described in other LAOs, which undergo deamination at the alpha ƒngroup. The only similar activity described so far is an LAO with a broad substrate range from *Rhodococcus* sp., which oxidizes the ɛ position of l-lysine when the alpha is blocked [134]. Thus, l-lysine-ɛ-oxidase synthesized by *M. mediterranea* received a new number from the Enzyme Commission (E.C. 1.4.3.20) [132].

(2) $$\text{L}-\text{lysine}+{\text{H}}_{2}\text{O}+{\text{O}}_{\text{2}}⇌2-\text{aminoadipate }6-\text{semialdehyde}+{\text{NH}}_{3}+{\text{H}}_{2}{\text{O}}_{2}$$

Another special characteristic of l-lysine-ɛ-oxidase is that it lacks the typical flavin coenzyme found in l-amino acid oxidases. Recent studies point to a quinonic cofactor at the catalytic site [135]. The gene coding for this activity was cloned and sequenced, and N-terminal sequencing of the purified protein, confirmed the correspondence between lysine oxidase activity and the gene *lodA* [133]. This gene is followed by another, *lodB*, in the same operon. Although the exact role of LodB has not been elucidated yet, both genes are essential for the expression of lysine oxidase activity [135]. While LodA is secreted to the extracellular medium during stationary phase of growth, LodB remains intracellular. Interestingly, this genomic organization as an operon is conserved in a series of homologous genes to *lodA* and *lodB* [133]. In one of those homologues to LodA, AlpP from the marine bacterium *Pseudoalteromonas tunicata*, the same l-lysine oxidase enzymatic activity was demonstrated [136].

As for the physiological function of l-lysine oxidase activity, it has been shown that *M. mediterranea* forms biofilms with a given architecture due to the autolytic effect of the l-lysine oxidase. As the microcolonies get thicker due to cell adhesion, the release of hydrogen peroxide as a consequence of the oxidation of l-lysine causes the death of a subpopulation of cells, which guaranties cell dispersal [136]. This phenomenon could be of great importance in the marine environment for *M. mediterranea*, which forms a part of the microbiota of the seagrass *Posidonia oceanica* [10]. The high molecular mass of LOD could prevent its dilution in aquatic media since it is retained in the polymeric matrix of the biofilm, as has been suggested for other large extracellular proteins [137]. Apart from that, it is tempting to speculate that the hydrogen peroxide released by lysine oxidase may not only play a role in biofilm dispersal but also in the competition between the colonizing microbiota of *Posidonia oceanica*.

## 5. Corollary: *M. mediterranea* as a Model Microorganism for the Study of Oxidase Activities

The study of the melanogenic bacterium *M. mediterranea* has revealed a unique set of properties. For instance, it was the first bacterium described expressing both laccase and tyrosinase activities [8]. The synthesis of melanins is considered an indication of a rich secondary metabolism related to the capacity to synthesize antibiotics [138]. In fact, *M. mediterranea* produces an antimicrobial molecule, but this is an LAO that oxidizes the amino acid l-lysine. Our studies have found that the expression of the three oxidases produced by *M. mediterranea* show some common characteristics. Firstly, all of them are growth phase regulated and are induced at the beginning of the stationary phase [139]. Secondly, the expression of these enzymes is regulated by the histidine quinase coded by *ppoS.* As shown in Figure 1, strain T103 mutated in *ppoS* does not synthesize melanins, which is a consequence of the decrease in tyrosinase levels. In this strain, a decrease in the laccase and LAO activities is also observed [133]. Despite the fact that the three oxidases show some common regulatory mechanisms, the analysis of mutants in individual oxidase genes has revealed that the mutation of a particular gene does not have any effect on the expression of the oxidases encoded by other genes [133]. The presence of common regulatory elements suggests that the three oxidases might be involved in the response to some environmental conditions. In this respect, analysis of the microbial genomes published revealed that the possession of genes encoding for the same three oxidases is uncommon. However, in the case of the epiphyte *Saccharophagus degradans* 2–40 [5] homologues to the three genes have been detected in consecutive order, suggesting that they form part of the same transcriptional unit, supporting the hypothesis of their relationship at the physiological level. As indicated above, LodA and similar proteins participate in biofilm development [136], so this might also be the case for the MCO and tyrosinase expressed by *M. mediterranea*.

Possession of genes encoding the three oxidases studied in *M. mediterranea* is uncommon even among species in the genus *Marinomonas*. In fact, the study of other *Marinomonas* species showed that only *M. mediterranea* expresses all three of them [10]. Moreover, analysis of the published genome of two *Marinomonas* strains (MWYL1 and MED121) confirms the absence of homologues to PpoA and PpoB. The draft sequencing, finishing, and annotation of *M. mediterranea* MMB-1 genome that will be done by the U.S. Department of Energy Joint Genome Institute will shed light on the origin of the unique genetic traits shown by this strain. So far, the study of this marine bacterium by a combination of physiological, biochemical and molecular means, has contributed to the detection of novel enzymes of possible biotechnological interest. Preliminary studies in our laboratory indicate that this microorganism expresses additional amino acid oxidases, raising intriguing questions concerning the extraordinary capacity of *M. mediterranea* to express diverse oxidases.
